# Geraniol Exerts Cytotoxic Effects in Red Cells Through Ca^2+^ Elevation and Membrane Hyperpolarization: Attenuating Effects of COX/CK1α/Rac1 GTPase Inhibition

**DOI:** 10.3390/molecules31101621

**Published:** 2026-05-12

**Authors:** Mohammad A. Alfhili, Shaymah H. Alruwaili, Jawaher Alsughayyir

**Affiliations:** Department of Clinical Laboratory Sciences, College of Applied Medical Sciences, King Saud University, Riyadh 12372, Saudi Arabia

**Keywords:** geraniol, hemolysis, eryptosis, calcium, anticancer

## Abstract

Background: Hemolysis and eryptosis of red blood cells (RBCs) contribute to chemotherapy-induced anemia, a marker of poor prognosis. Geraniol (GER) is an anticancer acyclic monoterpene alcohol found in several plant extracts, but a dearth of evidence exists regarding the potential toxicity of GER in RBCs. Methods: Hemolysis and eryptosis were evaluated using colorimetric and fluorescence-assisted cell-sorting methods, respectively. Phosphatidylserine (PS) exposure, loss of volume, and intracellular Ca^2+^ were measured by annexin-V-FITC, forward scatter (FSC), and Fluo4/AM staining. Cells were also examined by electron microscopy to identify membrane blebbing and by the Westergren method to assess erythrocyte sedimentation rate (ESR). Results: In a concentration-responsive fashion, GER induced hemolysis and PS exposure in addition to elevated ESR. GER-induced cell death was characterized by reduced FSC, membrane blebs, and increased Fluo4 fluorescence. Ca^2+^ deprivation prevented eryptosis, whereas concurrent Ca^2+^ deprivation and membrane depolarization prevented hemolysis, eryptosis, and cell shrinkage. Furthermore, whereas inhibition of cyclooxygenase (COX), casein kinase 1α (CK1α), or Rac1 GTPase ameliorated eryptosis and hemolysis, the latter was only prevented by caspase, nitric oxide synthase, or serine palmitoyltransferase inhibition. Exclusive reversal of eryptosis was rather only achieved in the presence of either caffeine or adenine. Conclusions: GER is a novel stimulator of hemolysis and eryptosis, an activity mediated through membrane hyperpolarization following Ca^2+^ elevation. In parallel, GER seems to involve the COX/CK1α/Rac1 GTPase axis to trigger its cytotoxic effects. Targeting the identified mechanisms in combination therapy may attenuate the off-target toxicity of GER and enhance its specificity to cancer cells.

## 1. Introduction

Geraniol (GER) is an acyclic monoterpene alcohol that is a constituent of the essential oils of numerous plants. The pharmacological properties of GER include antioxidant, anti-inflammatory, antimicrobial, and anticancer activities which have been demonstrated in vitro and in vivo [1,2]. Importantly, GER has been reported to be cytotoxic to prostate [3], colon [4], liver [5], lung [6], endometrial [7], thyroid [8], and nasopharyngeal [9] tumor cells. Numerous biochemical targets of GER have been revealed by these studies, identifying apoptosis as the major mechanism underlying its anticancer properties. Specifically, GER exposure leads to phosphatidylserine (PS) externalization; caspase stimulation; deceased cyclin D1, survivin, and Bcl2/Bax ratio; DNA damage; chromatin condensation; cell cycle arrest; increased reactive oxygen species (ROS); and Akt/mTOR inhibition.

Chemotherapy-related anemia is observed in up to 70% of cancer patients, and serves as an indicator of poor prognosis [10]. Hemolysis and eryptosis of red blood cells (RBCs) are recognized mechanisms underlying chemotherapy-related anemia [10,11] that could be exploited to improve patient outcomes. In this regard, early screening of emerging anticancer compounds for their non-specific toxicity, particularly in RBCs, may help develop strategies to mitigate their side effects.

Eryptosis is characterized by membrane blebbing and scrambling, PS exposure, Ca^2+^ elevation, oxidative stress, loss of volume, energy depletion, and ceramide formation. Major intracellular enzymes that regulate eryptosis include cyclooxygenase (COX), casein kinase 1α (CK1α), nitric oxide synthase (NOS), caspase, serine palmitoyltransferase (SPT), p38 MAPK, mixed lineage kinase domain-like (MLKL), and protein kinase C (PKC). While eryptosis serves as a protective mechanism by removing infected and damaged cells, complications of circulating eryptotic cells include vascular occlusion due to enhanced adherence to the endothelium. Increased eryptosis is also associated with a plethora of pathological conditions such as diabetes, hypertension, dyslipidemia, renal disease, and various forms of anemia, infections, and cancer [12].

Although GER has been demonstrated to reverse apoptosis in normal brain endothelial cells [13], a severe paucity still remains with regard to its cellular mechanisms in normal cells. This study aims to explore the potential cytotoxicity of GER in RBCs in an attempt to identify therapeutic targets to enhance its specificity to cancer cells.

## 2. Results

### 2.1. GER Is Cytotoxic to RBCs Through Hemolysis and Eryptosis

**Figure 1:**
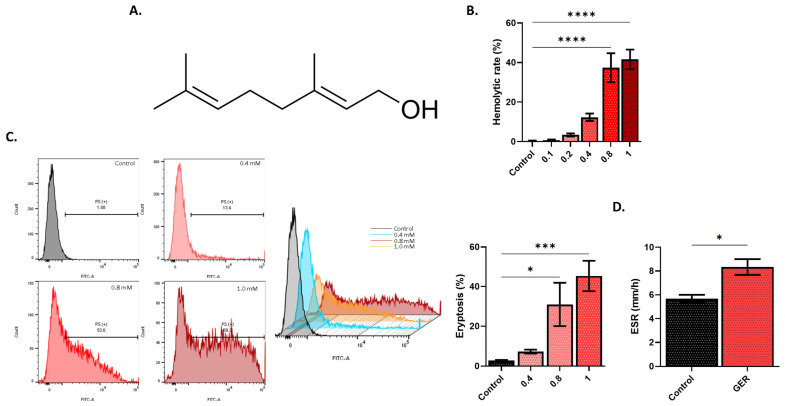
Cytotoxicity of GER. (**A**) Structure of GER, (**B**) hemolytic rate, (**C**) histograms of annexin-V-FITC and eryptosis, and (**D**) erythrocyte sedimentation rate (ESR) of control and experimental (0.8 mM of GER) cells. All experiments were run for 24 h at 37 °C and analyzed by one-way ANOVA, except for ESR, which was analyzed using the Student *t*-test (*n* = 9). * (*p* < 0.05), *** (*p* < 0.001), and **** (*p* < 0.0001).

Initially, experiments were conducted to explore whether GER is toxic to RBCs, and both hemolysis and eryptosis were assessed. GER significantly increased the hemolytic rate (*p* < 0.0001) and the percentages of eryptotic cells (*p* < 0.001) at 0.8 and 1.0 mM, as seen in Figure 1B and Figure 1C, respectively. Since eryptotic cells exhibit enhanced adherence to neighboring cells and tissues [12], the ESR was measured (Figure 1D) and was found to be significantly increased upon treatment with 0.6 mM of GER (*p* < 0.05).

### 2.2. GER Stimulates Ca^2+^ Elevation and Membrane Hyperpolarization

**Figure 2:**
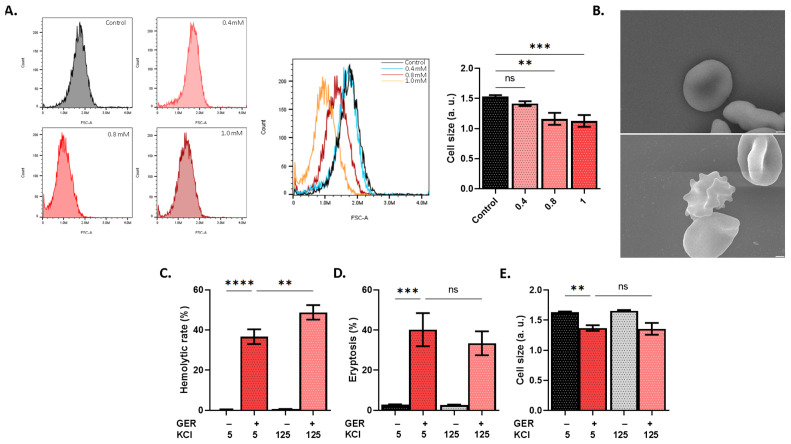
GER elicits membrane hyperpolarization and volume loss. (**A**) Histograms of forward scatter channel (FSC). (**B**) Membrane blebbing as visualized by electron microscopy (control and 0.8 mM of GER). Scale bar: 1 μm. Magnification: X7,000. Effects of membrane depolarization on (**C**) hemolytic rate, (**D**) eryptosis, and (**E**) cell size, as induced by 0.8 mM of GER. In panels (**C**–**E**), the first two bars show reference conditions while the last two bars show the corresponding experimental manipulation; “+” and “−” signs denote the presence and absence of the indicated factor, respectively. All experiments were run for 24 h at 37 °C and analyzed by one-way ANOVA (*n* = 9). No significance is shown by “ns”, whereas ** (*p* < 0.01), *** (*p* < 0.001), and **** (*p* < 0.0001).

Next, in order to reveal the mechanisms underlying the observed toxicity, we investigated the role of Ca^2+^ signaling and the ensuing cascade of events in GER-induced RBC death. Loss of volume is a downstream effect of Ca^2+^ entry, which leads to KCl and water efflux due to membrane hyperpolarization [12]. Congruently, GER significantly reduced cell size (*p* < 0.001; Figure 2A) and induced the appearance of membrane blebs (Figure 2B). Interestingly, suspending the cells in a medium containing 125 mM, as opposed to 5 mM, of KCl in order to induce membrane depolarization and block KCl and water efflux rather augmented hemolysis (*p* < 0.01; Figure 2C) and failed to reverse PS exposure (Figure 2D) or loss of volume (Figure 2E).

**Figure 3:**
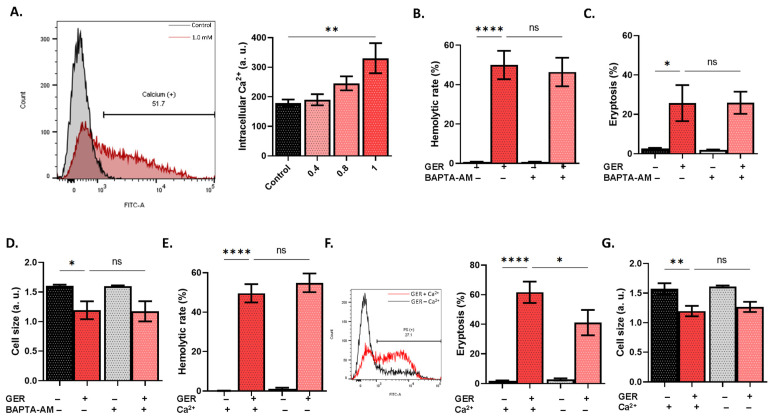
GER triggers Ca^2+^ elevation. (**A**) Histograms of Fluo4 fluorescence. Effects of BAPTA-AM on (**B**) hemolytic rate, (**C**) eryptosis, and (**D**) cell size, as induced by 0.8 mM of GER. Effects of extracellular Ca^2+^ deprivation on (**E**) hemolytic rate, (**F**) eryptosis, and (**G**) cell size, as induced by 0.8 mM of GER. In panels (**B**–**G**), the first two bars show reference conditions while the last two bars show the corresponding experimental manipulation; “+” and “−” signs denote the presence and absence of the indicated factor, respectively. All experiments were run for 24 h at 37 °C and analyzed by one-way ANOVA (*n* = 9). No significance is shown by “ns”, whereas * (*p* < 0.05), ** (*p* < 0.01), and **** (*p* < 0.0001).

While GER exposure results in significant Ca^2+^ elevation (*p* < 0.01; Figure 3A), chelation of intracellular Ca^2+^ using BAPTA-AM did not prevent the toxic effects of GER (Figure 3B–D). In contrast, GER exposure under extracellular Ca^2+^ deprivation, while not affecting hemolysis (Figure 3E), did nonetheless significantly prevent PS exposure (*p* < 0.05; Figure 3F). Cell size was, like hemolysis, not affected (Figure 3G).

**Figure 4:**
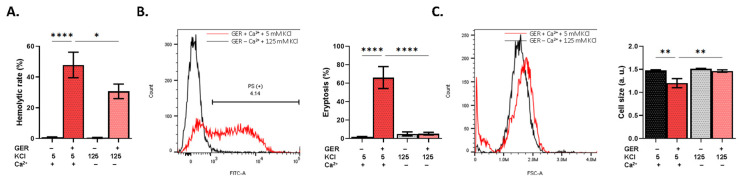
Ca^2+^ deprivation and membrane depolarization abrogate GER toxicity. (**A**) Hemolytic rate, (**B**) eryptosis, and (**C**) cell size, as induced by 0.8 mM of GER in the presence and absence of extracellular Ca^2+^ with 5 mM or 125 mM of KCl, respectively. The first two bars show reference conditions while the last two bars show the corresponding experimental manipulation; “+” and “−” signs denote the presence and absence of the indicated factor, respectively. All experiments were run for 24 h at 37 °C and analyzed by one-way ANOVA (*n* = 9). Whereas * (*p* < 0.05), ** (*p* < 0.01), and **** (*p* < 0.0001).

Given the observed modulation of ionic regulation by GER, we were prompted to examine the potential mitigating effect of the simultaneous elimination of extracellular Ca^2+^ and membrane depolarization. The results revealed that both manipulations significantly decreased the hemolytic rate (*p* < 0.05; Figure 4A) and abolished PS exposure (*p* < 0.0001; Figure 4B) and volume loss (*p* < 0.01; Figure 4C).

### 2.3. GER Cytotoxicity Is Independent of Oxidative Stress

**Figure 5:**
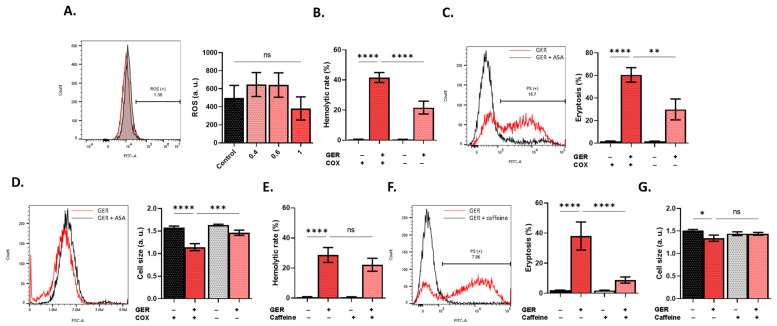
Protective effects of COX inhibition and caffeine against GER toxicity. (**A**) Histograms of dichlorofluorescein (DCF) fluorescence. Effects of acetylsalicylic acid (ASA) on (**B**) hemolytic rate, (**C**) eryptosis, and (**D**) cell size, as induced by 0.8 mM of GER. Effects of caffeine on (**E**) hemolytic rate, (**F**) eryptosis, and (**G**) cell size, as induced by 0.8 mM of GER. In panels (**B**–**G**), the first two bars show reference conditions while the last two bars show the corresponding experimental manipulation; “+” and “−” signs denote the presence and absence of the indicated factor, respectively. All experiments were run for 24 h at 37 °C and analyzed by one-way ANOVA (*n* = 9). No significance is shown by “ns”, whereas * (*p* < 0.05), ** (*p* < 0.01), *** (*p* < 0.001), and **** (*p* < 0.0001).

RBCs are particularly sensitive to oxidative stress, which constitutes a major mechanism underlying both hemolysis and eryptosis by opening Ca^2+^ channels [12]. However, intracellular ROS were not significantly increased upon GER treatment (Figure 5A). Despite an apparent lack of oxidative stress, Ca^2+^ channels may be activated by prostaglandin E_2_ which is formed by COX from arachidonic acid. Interestingly, COX inhibition by ASA significantly ameliorated hemolysis (*p* < 0.0001; Figure 5B), PS exposure (*p* < 0.01; Figure 5C), and cell shrinkage (*p* < 0.001; Figure 5D). Furthermore, caffeine, which inhibits Ca^2+^ influx [14], was not effective against hemolysis (Figure 5E), but, nonetheless, significantly decreased PS exposure (*p* < 0.0001; Figure 5F) with no effect on cell size (Figure 5G).

### 2.4. Inhibition of Rac1 GTPase and CK1α Attenuates GER Cytotoxicity

**Figure 6:**
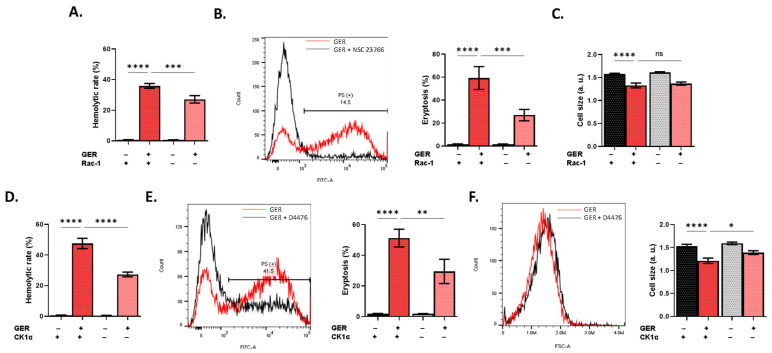
Rac1 GTPase and CK1α inhibition attenuates GER cytotoxicity. Effects of NSC 23766 on (**A**) hemolytic rate, (**B**) eryptosis, and (**C**) cell size, as induced by 0.8 mM of GER. Effects of D4476 on (**D**) hemolytic rate, (**E**) eryptosis, and (**F**) cell size, as induced by 0.8 mM of GER. The first two bars show reference conditions while the last two bars show the corresponding experimental manipulation; “+” and “−” signs denote the presence and absence of the indicated factor, respectively. All experiments were run for 24 h at 37 °C and analyzed by one-way ANOVA (*n* = 9). No significance is shown by “ns” whereas * (*p* < 0.05), ** (*p* < 0.01), *** (*p* < 0.001), and **** (*p* < 0.0001).

**Figure 7:**
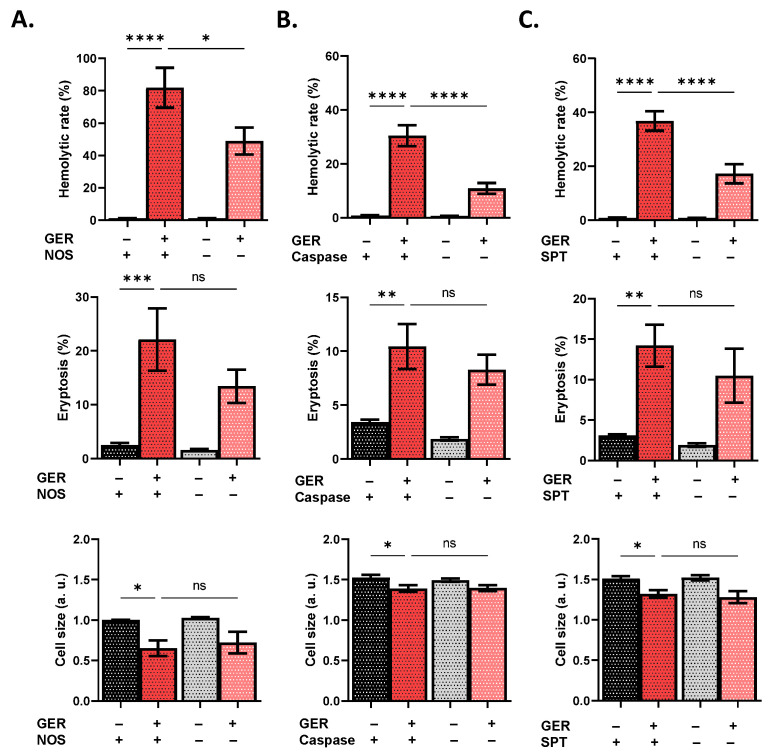
Inhibitors of GER-induced hemolysis. Effects of (**A**) L-NAME, (**B**) Z-VAD-FMK, and (**C**) myriocin on the hemolytic rate, eryptosis, and cell size, as induced by 0.8 mM (or 0.4 mM for eryptosis experiments) of GER. The first two bars show reference conditions while the last two bars show the corresponding experimental manipulation; “+” and “−” signs denote the presence and absence of the indicated factor, respectively. All experiments were run for 24 h at 37 °C and analyzed by one-way ANOVA (*n* = 9). No significance is shown by “ns”, whereas * (*p* < 0.05), ** (*p* < 0.01), *** (*p* < 0.001), and **** (*p* < 0.0001).

**Figure 8:**
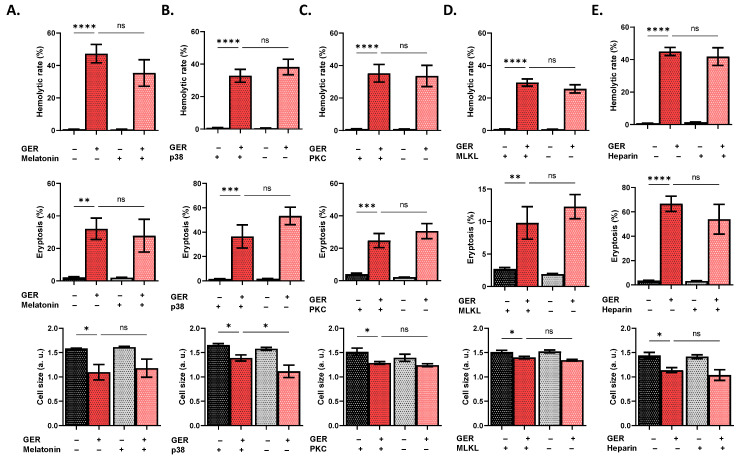
Mediators with no effect on GER toxicity. Effects of (**A**) melatonin, (**B**) SB203580, (**C**) staurosporin, (**D**) necrosulfonamide, and (**E**) heparin on the hemolytic rate, eryptosis, and cell size, as induced by 0.8 mM (or 0.4 mM under MLKL inhibition) of GER. The first two bars show reference conditions while the last two bars show the corresponding experimental manipulation; “+” and “−” signs denote the presence and absence of the indicated factor, respectively. All experiments were run for 24 h at 37 °C and analyzed by one-way ANOVA (*n* = 9). No significance is shown by “ns”, whereas * (*p* < 0.05), ** (*p* < 0.01), *** (*p* < 0.001), and **** (*p* < 0.0001).

In search of the signaling pathways mediating GER toxicity, we interrogated the participation of a wide spectrum of intracellular mediators as key modulators of RBC survival [12]. Using NSC 23766 to block Rac1 GTPase activity significantly reduced hemolysis (*p* < 0.001; Figure 6A) and eryptosis (*p* < 0.001; Figure 6B) but not cell shrinkage (Figure 6C). Moreover, inhibition of CK1α using D4476 significantly diminished hemolysis (*p* < 0.0001; Figure 6D), eryptosis (*p* < 0.01; Figure 6E), and shrinkage (*p* < 0.05; Figure 6F). Additionally, GER-induced hemolysis, but not eryptosis, was significantly decreased in the presence of L-NAME (*p* < 0.05), Z-VAD-FMK (*p* < 0.0001), and myriocin (*p* < 0.0001), as seen in Figure 7A, 7B, and 7C, respectively. Notably, Figure 8A–E demonstrate interventions that failed to reverse any toxic endpoint.

### 2.5. Effect of Metabolic Substrates on GER Cytotoxicity

**Figure 9:**
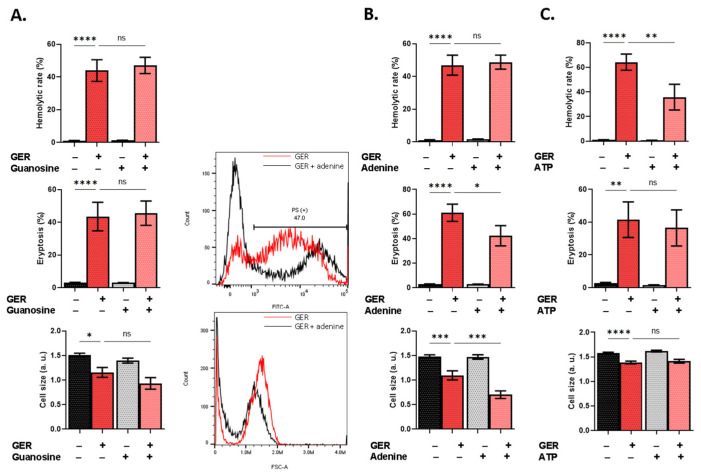
Effect of energy substrates on GER toxicity. Effects of (**A**) guanosine, (**B**) adenine, and (**C**) ATP on the hemolytic rate, eryptosis, and cell size, as induced by 0.8 mM of GER. The first two bars show reference conditions while the last two bars show the corresponding experimental manipulation; “+” and “−” signs denote the presence and absence of the indicated factor, respectively. All experiments were run for 24 h at 37°C and analyzed by one-way ANOVA (*n* = 9). No significance is shown by “ns”, whereas * (*p* < 0.05), ** (*p* < 0.01), *** (*p* < 0.001), and **** (*p* < 0.0001).

We also explored the protective properties of metabolic substrates against GER toxicity, since energy depletion precedes RBC death [12]. While guanosine (Figure 9A) offered no protection, adenine significantly diminished PS exposure (*p* < 0.05) but exacerbated volume loss (*p* < 0.001). ATP, on the other hand, only reversed hemolysis (*p* < 0.01; Figure 9C).

## 3. Discussion

The current study explores the hematological toxicity of GER and uncovers that it exerts cytotoxic effects in RBCs through eryptosis and hemolysis. This nonspecific toxicity was observed at concentrations that overlap with the range in which GER shows anticancer properties. While this undermines the clinical prospects of GER, combination therapy may be able to overcome this limitation by modulating the mechanisms activated by GER in RBCs.

Hemolysis (Figure 1) is an accidental type of cell death that invariably leads to hemoglobin precipitation in the kidneys, giving rise to intratubular hemoglobin casts and loss of renal function [15]. Importantly, GER-induced hemolysis was significantly ameliorated by blocking NOS, caspases, and SPT activities (Figure 7). Inhibition of NOS using L-NAME was reported to attenuate the ROS and PS externalization elicited by cholestan-3β,5α,6β-triol [16]. Very recently, Sangha et al. [17] demonstrated that NOS phosphorylation in response to stimulation by the Piezo1 Ca^2+^ channel is augmented in diabetic RBCs compared to those from healthy subjects. Furthermore, caspases were involved in RBC death caused by ferutinin [18], and the crosstalk between caspases and ceramide formation, which occurs through SPT, was elucidated in erythrocytes exposed to cigarette smoke extract, the effects of which also include Ca^2+^ elevation (Figure 3) [19]. Indeed, drugs may cause hemolytic anemia through antibody formation, and whether GER is capable of eliciting an immune response remains to be elucidated in future in vivo studies. Altogether, the findings regarding hemolysis propose the investigation of these pathways and their respective modulators as potential targets to optimize the clinical utility of GER.

Translocation of PS to the outer membrane leaflet (Figure 1) is pathognomonic of eryptosis and primes dead cells for uptake and degradation by phagocytes. Indeed, the prolonged presence of dysfunctional cells in the circulation carries the risk of thrombosis since PS-exposing cells show stronger adherence to endothelial tissue through CXCL16/SR-PSOX [20]. Concordantly, the current results reveal that ESR is elevated in GER-treated cells (Figure 1). Although not a marker of endothelial adhesion, ESR is a physical marker of RBC aggregation that could be influenced by plasma proteins (32491417). However, in the current study, we measured ESR in isolated RBCs, thereby significantly minimizing the potential confounding effects of plasma proteins. As such, the observed elevation in ESR seems to indicate GER-induced altered surface charges conducive to cell–cell interactions that may promote adhesive behavior in vivo. This interpretation is supported by evidence from clinical studies demonstrating significant positive correlations between ESR and adhesion molecules such as VCAM-1, ICAM-1, and E-selectin (34078723, 12022343, 19578810). Conversely, decreases in erythrocyte count and their oxygen-carrying capacity are expected if the rate of eryptosis exceeds the capacity of the bone marrow to replenish the pool of healthy circulating RBCs. As such, the use of erythropoiesis-stimulating agents alongside GER may aid in mitigating its adverse effects on RBCs.

Notably, GER exposure is accompanied by Ca^2+^ entry from the extracellular space through Ca^2+^-permeable cation channels, which in turn activates Ca^2+^-sensitive K^+^ channels (Gardos) to mediate the loss of KCl and water. Collectively, this gives rise to the typical biochemical features of eryptotic cells, including loss of volume (Figure 2) downstream of intracellular Ca^2+^ elevation (Figure 3). Interestingly, the mechanistic distinction between hemolysis and eryptosis induced by GER is revealed by the fact that Ca^2+^ influx is important to the latter but not to the former (Figure 3). Also, membrane depolarization alone failed to inhibit hemolysis or eryptosis (Figure 2). Rather, Ca^2+^ entry and the ensuing membrane hyperpolarization both seem to be required for either hemolysis or eryptosis and volume loss to occur (Figure 4). These observations suggest that Ca^2+^ is perhaps the most important regulator of GER-induced RBC death, identifying it as a novel mediator of GER toxicity and further cementing its role in RBC physiology [21]. Along those lines, the Gardos channel inhibitor senicapoc, which prevents cell dehydration by blocking KCl release, has been successfully used to manage sickle cell disease and other conditions [22,23].

In this work, ROS levels remained unchanged upon GER treatment (Figure 5), which is in agreement with its reported function as an antioxidant [1,2]. However, it has also been reported that GER increases ROS in liver and thyroid cells [5,8], decreases total antioxidant capacity in lung cancer cells [6], and reduces glutathione and superoxide dismutase activity in nasopharyngeal carcinoma cells [9]. Thus, the impact of GER on redox balance appears to be context-specific, and does not seem to be of relevance in RBCs under the current experimental conditions.

It must be noted that pharmacological inhibition identifies the pathway requirement to the tested endpoint but does not necessarily establish direct molecular targeting. As such, the results of functional assays using enzyme inhibitors are interpreted as associations rather than definitive mechanistic evidence. Earlier studies demonstrated that COX is a key player in the mechanisms leading up to RBC death, including osmotic challenge and chloride deprivation [24]. Congruently, the current rescue experiments revealed that blocking COX activity using ASA rescues the cells from GER-induced hemolysis, PS exposure, and cell shrinkage (Figure 5). The function of this enzyme involves the conversion of arachidonic acid to prostaglandin E_2_, an inflammatory mediator that has been shown to promote Ca^2+^ influx (Figure 3). Taken together, these results suggest that GER seems to stimulate COX to generate prostaglandin E_2_, which acts upstream of Ca^2+^ elevation. This is further supported by the results involving caffeine supplementation, which alleviated PS exposure (Figure 5), possibly by modulating Ca^2+^ availability [14].

Other enzymes that appear to be involved in GER toxicity include Rac1 GTPase and CK1α (Figure 6). To date, very little is known about the function of Rac1 in RBCs. In murine erythrocytes, Rac1 preserves the cytoskeletal architecture and prevents sickling [25], while later investigations have revealed its role in mediating human erythrocyte death triggered by oxysterol [16] and galangin [26], among other compounds. Rac1 has also been reported to facilitate parasitic invasion of cells by *P. falciparum* [27], arguing for its dual role, a prospect which requires further clarification. This is in contrast to CK1α, which has, comparatively, been more extensively studied in RBCs. Activation of this kinase stimulates RBC death by a variety of xenobiotics and adverse physiological conditions such as oxidative stress, Ca^2+^ elevation, and metabolic starvation [28]. Taking into consideration the reversal of PS exposure under Ca^2+^ elimination (Figure 3) and in the presence of adenine (Figure 9), the current findings collectively seem to suggest that the increase in cytosolic Ca^2+^ by GER depletes energy stores, which then may activate CK1α. As such, D4476 presents itself as a potential lead compound for counteracting the toxicity of GER in RBCs.

In conclusion, this study provides evidence of the nonspecific cytotoxicity of GER and reveals its hemolytic and eryptotic activities, along with an extensive set of molecular mechanisms. These include membrane injury; modulation of membrane phospholipids; disruption of Ca^2+^ and K^+^ trafficking; loss of volume; membrane blebbing; inflammatory phenotype suggestive of enhanced aggregation; and the participation of a complex cascade potentially involving COX, Rac1, CK1α, caspase, NOS, and SPT. These findings seem to implicate prostaglandin E_2_, nitric oxide, and ceramide in GER erythrotoxicity and open the door for pharmacological interventions to improve the therapeutic prospects of GER in anticancer therapy.

## 4. Materials and Methods

### 4.1. RBC Purification

Experiments were conducted according to the guidelines of the Eryptosis Study Consortium [12]. This work was undertaken with ethical approval from the IRB committee of King Saud University (E-24-8762) and in accordance with the Helsinki Declaration. Blood samples were taken in lithium heparin vacutainer tubes, with informed consent, from fourteen participants. The subjects were nine males and five females, with an age range of 27 to 39 years, free from acute or chronic conditions, and nonsmoking as assessed by verbal confirmation. All subjects had normal complete blood count results. Whole blood aliquots were centrifuged at 2500 rpm for 15 min and washed twice in physiological saline to discard the plasma and buffy coat. Pure RBC suspensions were prepared in Ca^2+^-free Ringer buffer and stored at 4 °C for no more than 24 h [12].

### 4.2. Experimental Design

All kits and chemicals were provided by Solarbio Life Sciences (Beijing, China). A 100 mM (15.42 mg/mL) stock solution of GER was prepared and stored at –80 °C. RBCs were suspended in a standard Ringer buffer consisting of 125 mM of NaCl, 5 mM of KCl, 1 mM of MgSO_4_, 1 mM of CaCl_2_, 32 mM of HEPES, and 5 mM of glucose (pH = 7.4). Cells were treated with 0.1, 0.2, 0.4, 0.8, and 1.0 mM of GER for 24 h at 37 °C. This range was chosen in order to mimic the anticancer concentrations previously reported for GER [3,6,7]. All experiments included a negative control consisting of cells exposed to 1% DMSO, corresponding to the respective amount in the highest GER concentration used, and a positive control prepared by suspending cells in distilled water instead of standard Ringer buffer. Manipulations of standard Ringer buffer included the addition of 125 mM of KCl instead of 5 mM, and Ca^2+^ omission. Functional assays were performed using a wide range of physiological modulators added to standard Ringer buffer with and without 0.8 mM of GER. Due to specific drug–drug interactions, 0.4 mM of GER was used instead in some eryptosis experiments. These included the cell-permeable Ca^2+^ chelator BAPTA-AM (20 μM), COX inhibitor acetylsalicylic acid (ASA; 50 μM), caffeine (0.5 mM), Rac1 GTPase inhibitor NSC 23766 (μM), CK1α (20 μM), NOS inhibitor L-NAME (20 μM), pan-caspase inhibitor Z-VAD-FMK (100 μM), SPT inhibitor myriocin (10 μM), melatonin (50 μM), p38 MAPK inhibitor SB203580 (100 μM), PKC inhibitor staurosporin (1 μM), MLKL inhibitor necrosulfonamide (0.5 μM), heparin (1 U/mL), guanosine (2 mM), adenine (2 mM), and ATP (0.5 mM) [12]. In each experiment, the first two bars represent the reference (control) conditions of standard Ringer buffer while the last two bars represent the corresponding experimental intervention. The use of “+” and “−” signs denotes the nature of the reference condition, which may indicate the presence of physiological factors (e.g., extracellular Ca^2+^) or the absence of added exogenous compounds (e.g., modulators). This presentation convention is consistently followed across all experiments.

### 4.3. Hemolysis

The relative amount of hemoglobin in the supernatant was measured at OD_405_ using a plate reader (LMPR-A14, Labtron, Camberley, Surry, UK) [12].

### 4.4. Eryptosis

Eryptosis was assayed by flow cytometry (Cytek Biosciences, Fremont, CA, USA). PS exposure was quantified using annexin-V-FITC. Cells were suspended in annexin-binding buffer containing 1% annexin-V-FTIC for 10 min at room temperature in the dark. Using light intensity captured in the forward scatter channel (FSC), loss of volume was assessed. Intracellular Ca^2+^ (5 μM of Fluo4/AM in annexin-binding buffer) and ROS (10 μM of H_2_DCFDA in Ringer buffer) were also measured following incubation of cells for 30 min at 37 °C in the dark. All dyes were excited by the blue laser at 480 nm to emit green light, which was detected at 520 nm. All readings were recorded for 10,000 cells [12].

### 4.5. Erythrocyte Sedimentation Rate (ESR)

The ESR was measured as a marker of RBC aggregation in washed suspensions, using the Westergren method [12].

### 4.6. Electron Microscopy

Membrane blebs were visualized using a scanning electron microscope (JSM-7610F, JEOL, Tokyo, Japan) as per standard protocol [12].

### 4.7. Statistics

Data were analyzed by one-way ANOVA (corrected by Dunnett’s test) or Student *t*-test using GraphPad Prism 9.0. All experiments were run three times each with three technical replicates for a total of nine readings per condition. Figures show histograms as means and error bars as standard error of the mean (SEM). The calculated difference in variance among experimental conditions was considered statistically significant only if an error rate of at least <0.05 was achieved.

## Data Availability

The data presented in this study are available on request from the corresponding author. The data are not publicly available due to privacy and ethical restrictions.
